# Financial toxicity amongst cancer patients and survivors: a comparative study of the United Kingdom and United States

**DOI:** 10.1007/s00520-025-09568-6

**Published:** 2025-05-31

**Authors:** Tran Thu Ngan, Emily Tonorezos, Michael Donnelly, Ciaran O’Neill

**Affiliations:** 1 Centre for Public Health, School of Medicine, Dentistry and Biomedical Sciences, Queen’s University Belfast, Belfast, UK; 2 Office of Cancer Survivorship, Division of Cancer Control and Population Sciences, National Cancer Institute, Rockville, MD USA

**Keywords:** Financial toxicity, Cancer, Cancer survivorship, COmprehensive Score for financial Toxicity, Universal health coverage

## Abstract

**Background:**

The study investigated the experiences of financial toxicity (FT) amongst cancer patients/survivors in the United Kingdom (UK) and the United States (USA/US).

**Methods:**

Six hundred cancer patients/survivors residing in the UK (*n* = 319) or USA/US (*n* = 281) completed an online cross-sectional survey using the COmprehensive Score for financial Toxicity (COST)—a validated measure of FT. Severity of FT was defined as ‘no’ (COST scores ≥ 26), ‘mild’ (14–25), and ‘moderate/severe’ (0–13).

**Results:**

Thirty-four percent of UK participants faced FT which was significantly lower compared to the USA/US at 55% (crude OR = 2.44, 95% CI 1.73–3.42). An ordered logistic regression model showed that in the USA/US, being 65 + years old (adjusted OR = 0.19, 95% CI 0.07–0.48), retired (aOR = 0.26, 95% CI 0.09–0.75), and having a higher household income (aOR ranged 0.03–0.19) decreased the risk of FT, whilst being female increased the risk (aOR = 1.83, 95% CI 1.01–3.32). In the UK, age and sex did not have an effect, but higher income and being retired showed an identical pattern compared to the US.

**Conclusions:**

FT was less prevalent and less severe in the UK, compared to the USA/US. The high prevalence of FT underscores the need to provide an additional level of protection to the most vulnerable groups than is currently offered in either country.

**Supplementary Information:**

The online version contains supplementary material available at 10.1007/s00520-025-09568-6.

## Background

The term ‘financial toxicity’ (FT), proposed by Zafar and Abernethy (2013), refers to the objective financial burden and subjective financial distress that can attend a cancer diagnosis and treatment [1, 2]. Research has largely focused on the material consequences of treatment by quantifying the objective financial cost rather than the psychosocial impact of treatment costs [2, 3]. People diagnosed with cancer and faced with FT may experience worse financial well-being, lower health-related quality of life (HRQoL), and additional mental health problems [4–8]. The term FT, therefore, is used to emphasise the adverse consequences of financial stress on health outcomes and to convey a stronger sense of urgency and importance to policy makers and practitioners around the issue than might otherwise be engendered.

Around 70% of studies on FT come from the United States (USA/US) [9] where publicly financed and privately financed health coverage coexist. Recent studies from Canada, Italy, Germany, and Japan have shown that FT is common even in countries with publicly funded healthcare system [5, 10–12]. In these countries as well as in the United Kingdom (UK), whilst healthcare services are provided free at the point of use, other direct non-medical and indirect costs can still fall on patients. The risk of FT can therefore arise in these systems with the risk and level of severity varying, in part, by differences in access to publicly funded care, welfare supports, and other healthcare inequalities. In this context, a comparative study between two countries with different healthcare funding like the UK and USA/US may shed light on the experience of FT.

In the UK, no study has investigated FT according to the encompassing definition of FT (discussed above) or used a validated measure of FT [13]. The majority of studies that have been conducted have focused on describing the objective financial burden and its material impact on the financial well-being of cancer patients, survivors, and/or carers/family members. Subjective financial distress, especially the psychosocial effects, is under-researched [13]. Moreover, inadequate research attention has been paid to the investigation of disparities amongst different sociodemographic groups of FT [13]. These critical research gaps may undermine the case for FT to be given due priority amongst policy makers or for feasible solutions to be identified and developed.

It is hypothesised that the prevalence of FT is (i) higher in the USA/US compared to UK due to the difference in financing mechanism of the two health systems and (ii) varies amongst those with different socio-demographic and/or severity of cancer treatment. Therefore, the study aims to investigate the prevalence of FT amongst cancer patients and survivors in the UK and US and assess the nature of FT including its severity and distribution across groups with different sociodemographic and clinical-related cancer characteristics.

## Methods

### Study design and participants

An online cross-sectional study was conducted in February 2023, wherein respondents were identified and recruited from Prolific (https://www.prolific.co/), a research platform that enables researchers to recruit and collect data from registered members. Prolific has been used in previous cancer survivorship research [14, 15]. Recent study also found that Prolific participants produced higher quality data and are more diverse than other crowdsourcing platforms [16].

Inclusion criteria for participants were (i) 18 + years old, (ii) residing in the UK or USA/US, and (iii) have or had cancer regardless of the types. Sample size was calculated according to World Health Organization guidance and using a formula that estimated a population proportion with specific absolute precision [17]. The estimated proportion of targeted participants who experience FT was assumed at 50% to generate the most conservative (i.e. largest) sample size. A sample size of 600 was calculated using a 95% confidence interval, an absolute precision value of 0.1, a design effect of two (to account for convenience sampling), and a non-response rate of 45%.

Prolific sent an invitation letter (prepared by the author) and survey link to all eligible members by comparing the study’s inclusion criteria with pre-existing members’ profile, comprised of more than 250 + characteristics completed by members at the time of registration with Prolific (e.g. if one stated that they did not have cancer at the time of completing their profile for registration, they could not receive the survey invitation). Eligible participants could access the survey link on a first come first served basis until the predetermined sample size was reached. To minimise the effect of rapid-responder bias, reach a wider audience, and accommodate the different time zones, the survey was paused five times and restarted at different time of the day.

The study was carried out in accordance with the ethical standards of the Helsinki Declaration. It received the ethical approval no. MHLS 22_178 dated 18 th January 2023 from Faculty of Medicine, Health and Life Sciences Research Ethics Committee, Queen’s University Belfast, UK. Informed consent was obtained from all individual participants included in the study. Participants were compensated £2.40 for their time upon completion of the survey.

### Variables and measurements

#### Main outcome: financial toxicity

The validated 12-item COmprehensive Score for financial Toxicity (COST) instrument [18, 19] was used to measure FT. The COST instrument was chosen because of its extensive use internationally, and its use enhanced the opportunities to compare results with other studies. The COST score ranges from 0 to 44 with three grading scale (proposed by COST’s authors) of ‘no FT’ (COST ≥ 26), ‘mild FT’ (COST 14–25), and ‘moderate/severe FT’ (COST 0–13) [19]. COST items and scoring guidelines are presented in Supplementary Table 1.

#### Covariates

The sociodemographic and clinical-related risk factors of FT used in descriptive and bivariate analysis were based on previous systematic reviews [8, 20–22]. The sociodemographic characteristics considered were ‘gender’ (self-report, derived from the question ‘Which of the following best describes you?’ with options of male, female, and others), ‘age group’ (18–64 vs 65 +), ‘education’, ‘marital status’, ‘occupation’, ‘main earner status’ (Yes/No), and ‘household weekly income’ (at time of survey). The clinical characteristics considered were ‘stage of cancer at diagnosis’, ‘treatment status’ (on-treatment, off-treatment, or expected to be on-treatment for the remainder of life), and ‘time since diagnosis’ (≤ 1 year, > 1 year).

#### Statistical analysis

Descriptive statistics (mean and standard deviation (SD) for continuous variables, proportions for discrete variables) were used to describe the socio-demographic and clinical-related characteristics of the participants as well as their FT severity. Differences in FT severity between participants living in the USA/US or UK were tested using chi-square/Fisher’s exact test. In multivariate analyses, the factors that influence FT severity were investigated using two ordered logistic models, one for participants from each country. First, all mentioned covariates were included in the models. Then, using a backwards elimination approach and Akaike + Bayesian information criteria (AIC and BIC for model goodness-of-fit), a final model was formed. Tests of statistical significance are two-sided; the cut-off for statistical significance was 0.05. All statistical procedures were conducted in STATA 15.0.

## Results

### Characteristics of study participants

The online survey was run on Prolific platform from 15 to 24 February 2023 (I WAS ended automatically when it reached the predetermined sample size). During this period, 639 individuals clicked the survey link, 626 (98%) consented, and 600 (response rate = 94%) completed the survey (UK = 319 (53%) and USA/US = 281 (47%)). The participants came from all 12 regions of UK and 46/50 states of USA/US (Supplementary Table 2).

Table 1 presents the sociodemographic and cancer-related characteristics of participants. The mean age of participants was 52 (sd = 14, range = 21–93). Female participants accounted for 66.5%; 42.5% participants had breast cancer, cervical cancer, ovarian cancer, or uterine and endometrial cancer (Supplementary Table 3). Approximately half of the participants were married (54.5%) and the main earner of the household (52.7%). Regarding occupation, 35.3% participants worked full-time, 14.2% worked part-time, 21% were retired, and 12.2% were self-employed. There were no significant differences between participants from the USA/US and UK regarding the above sociodemographic characteristics.

**Table 1:** Sociodemographic and cancer-related characteristics of participants

	Total; *n* (%)	UK; *n* (%)	USA/US; *n* (%)	*p*-value*
Total	600 (100.0)	319 (53.2)	281 (46.8)	
Age, mean (SD)	52 (14)	53 (14)	51 (14)	NS
Age groups				
18–64 years old	180 (80.0)	255 (80.0)	225 (80.0)	NS
65 + years old	120 (20.0)	64 (20.0)	56 (20.0)	
Gender				
Male	197 (32.8)	108 (33.9)	89 (31.7)	NS
Female	399 (66.5)	211 (66.1)	188 (66.9)	
Education				
Graduate degree or above	55 (9.2)	49 (15.3)	6 (2.2)	< 0.001
Undergraduate degree	87 (14.5)	46 (14.4)	41 (14.6)	
Technical/community college	111 (18.5)	57 (17.9)	54 (19.2)	
High school diploma/A-levels	221 (36.8)	107 (33.5)	114 (40.6)	
Secondary education	109 (18.2)	52 (16.3)	57 (20.3)	
No formal qualifications	17 (2.8)	8 (2.5)	9 (3.2)	
Marital status				
Single/never married	90 (15.0)	45 (14.1)	45 (16.0)	NS
Married	327 (54.5)	177 (55.5)	150 (53.4)	
In a relationship	86 (14.3)	55 (17.2)	31 (11.0)	
Divorced or separated	74 (12.3)	31 (9.7)	43 (15.3)	
Widowed	22 (3.7)	11 (3.4)	11 (3.9)	
Occupational status				
Employed full-time	212 (35.3)	109 (34.2)	103 (36.7)	NS
Employed part-time	85 (14.2)	48 (15.0)	37 (13.2)	
Unemployed	24 (4.0)	10 (3.1)	14 (5.0)	
Self-employed	73 (12.2)	36 (11.3)	37 (13.2)	
Full-time homemaker	21 (3.5)	8 (2.5)	13 (4.6)	
Retired	126 (21.0)	78 (24.5)	48 (17.1)	
Still studying	10 (1.7)	3 (0.9)	7 (2.5)	
Disabled/too ill to work	49 (8.2)	27 (8.5)	22 (7.8)	
Being main earner of household	316 (52.7)	166 (52.0)	150 (53.4)	NS
Household weekly income, before tax				
Up to £200 (up to $250)	45 (7.5)	22 (6.9)	23 (8.2)	< 0.001
£200–399 ($250–499)	85 (14.2)	52 (16.3)	33 (11.7)	
£400–599 ($500–749)	114 (19.0)	70 (21.9)	44 (15.7)	
£600–799 ($750–999)	89 (14.8)	51 (16.0)	38 (13.5)	
£800–999 ($1000–1249)	66 (11.0)	44 (13.8)	22 (7.8)	
£1000–1199 ($1250–1499)	48 (8.0)	27 (8.5)	21 (7.5)	
£1200–1399 ($1500–1749)	41 (6.8)	19 (6.0)	22 (7.8)	
£1400 or above ($1750 or above)	94 (18.8)	34 (10.6)	78 (27.7)	
Stage of cancer at diagnosis				
Stage 0 (carcinoma in situ)	76 (12.7)	33 (10.3)	43 (15.3)	0.03
Stage I	145 (24.2)	67 (21.0)	78 (27.8)	
Stage II	132 (22.0)	73 (22.9)	59 (21.0)	
Stage III	91 (15.2)	55 (17.2)	36 (12.8)	
Stage IV (metastatic)	44 (7.3)	27 (8.5)	17 (6.0)	
Treatment status				
Off-treatment (in remission/N.E.D)	465 (77.5)	247 (77.4)	218 (77.6)	NS
On-treatment	88 (14.7)	50 (15.7)	38 (13.5)	
Expected to be on-treatment for the remainder of life	47 (7.8)	22 (6.9)	25 (8.9)	
Time since cancer diagnosis				
≤ 1 year	56 (9.5)	25 (8.0)	31 (11.3)	NS
> 1 year	533 (90.5)	289 (92.0)	244 (88.7)	

*N.E.D* no evidence of disease state, *NS* not significant, *SD* standard deviation

^*^Results of chi-square tests compared between participants in the UK vs the USA/US

The majority of participants were White (88%); nearly two thirds had at least high school diploma (57.8%), a weekly household income under £999 or $1249 (66.5%), and were diagnosed at early stages (stage 0/I/II) of cancer (58.9%). Regarding treatment status, 77.5% of participants were off-treatment (i.e. in remission/N.E.D state), 14.7% were on-treatment, and 7.8% were expected to be on-treatment for the remainder of life.

### Prevalence of FT in the USA/US & UK

**Fig. 1:**
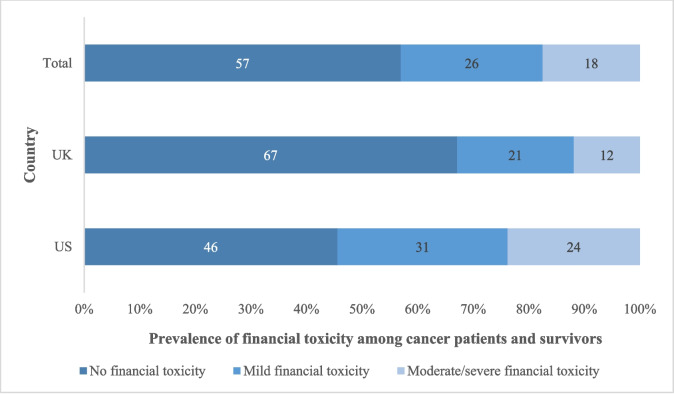
Prevalence of financial toxicity by severity level and country

The average COST scores were 28.7 (SD = 10.6) and 23.2 (SD = 11.0) in the UK and USA/US, respectively. In the UK, 21% and 12% of participants faced mild and moderate/severe FT, respectively (Fig. 1). In the USA/US, the prevalence was significantly higher at 31% and 24%, respectively (*p* < 0.001). The odds that a USA/US participant had FT was 2.44 times higher than the odds of a UK participant (crude OR = 2.44, 95% CI 1.73–3.42).

### Distribution of FT among sociodemographic groups in the USA/US & UK

Table 2 presents the distribution of FT amongst groups with different sociodemographic and clinical characteristics. The prevalence of moderate/severe FT was highest amongst participants who expected to be on-treatment for the remainder of life, followed by the on-treatment group, and lowest in the off-treatment group (*p* = 0.002). This descending pattern was observed in both the USA/US (68%, 61%, and 52%, respectively) and UK (64%, 34%, and 30%, respectively). The differences were significant amongst the group who were on-treatment and off-treatment (*p* = 0.02, Fisher’s exact test and *p* < 0.0001, chi-square test, respectively) but was not statistically significant amongst the group of participants expected to be on-treatment for the remainder of life.

**Table 2:** Distribution of financial toxicity amongst groups with different sociodemographic and clinical characteristics by country

	*n* (%) of individuals having FT*		*p*-value**
	UK	USA/US	
Age groups			
18–64 years old	95 (37.3)	142 (63.1)	< 0.001
65 + years old	10 (15.6)	11 (19.6)	NS
Gender			
Male	31 (28.7)	37 (41.6)	NS
Female	74 (35.1)	113 (60.1)	< 0.001
Occupational status			
Employed full-time	29 (26.6)	53 (51.5)	< 0.001
Employed part-time	16 (33.3)	28 (75.7)	< 0.001
Unemployed	5 (50.0)	10 (71.4)	NS
Self-employed	19 (52.8)	18 (48.6)	NS
Full-time homemaker	4 (50.0)	9 (69.2)	NS
Retired	8 (10.3)	10 (20.8)	NS
Still studying	1 (33.3)	5 (71.4)	NS
Disabled/too ill to work	23 (85.2)	20 (90.9)	NS
Main earner of household			
No	59 (38.6)	77 (58.8)	0.001
Yes	46 (27.7)	76 (50.7)	< 0.001
Household weekly income, before tax			
Up to £200 (up to $250)	13 (59.1)	20 (87.0)	0.037
£200–399 ($250–499)	26 (50.0)	20 (60.6)	NS
£400–599 ($500–749)	24 (34.3)	29 (65.9)	0.001
£600–799 ($750–999)	18 (35.3)	20 (52.6)	NS
£800––999 ($1000–1249)	4 (9.1)	13 (59.1)	< 0.001
£1000–1199 ($1250–1499)	8 (29.6)	15 (71.4)	0.005
£1200–1399 ($1500–1749)	5 (26.3)	6 (27.3)	NS
£1400 and above ($1750 and above)	7 (20.6)	30 (38.5)	0.049
Stage of cancer at diagnosis			
Stage 0 (carcinoma in situ)	7 (21.2)	18 (41.9)	0.048
Stage I	18 (26.9)	36 (46.2)	0.013
Stage II	22 (30.1)	40 (67.8)	< 0.001
Stage III	25 (45.5)	21 (58.3)	NS
Stage IV (metastatic)	14 (51.9)	14 (82.4)	0.040
Treatment status			
Off-treatment (in remission/N.E.D)	74 (30.0)	113 (51.8)	< 0.001
On-treatment	17 (34.0)	23 (60.5)	0.012
Expected to be on-treatment for the remainder of life	14 (63.6)	17 (68.0)	NS
Time since cancer diagnosis			
≤ 1 year	10 (40.0)	16 (51.6)	NS
> 1 year	93 (32.2)	135 (55.3)	NS

*COST* COmprehensive Score for financial Toxicity instrument, *N.E.D* no evidence of disease state, *NS* not significant, *UK* the United Kingdom, *USA/US* the United States

^*^Based on COST score (potential range: 0–44): individuals were categorised as ‘having FT’ if their COST score < 26

^**^Results of Fisher’s exact tests compared between participants in the UK and the USA/US

The proportion of USA/US participants who were 18–64 years old and facing FT was 1.7 times higher than the same group in the UK (63% vs 37%, respectively; *p* < 0.001). This difference was not seen in those 65 + (20% vs 16%, respectively; *p* = 0.563).

FT prevalence was two times higher in the USA/US compared to the UK amongst those who were employed full-time (52% vs 27%, respectively) or part-time (76% vs 33%, respectively) (*p* < 0.001, Fisher’s exact tests). There was no difference between the USA/US and UK in the prevalence of FT amongst those were self-employed or retired.

Results of the ordered logistic models investigating FT and its associated factors are presented in Tables 3 (UK case) and 4 (USA/US case). In the UK, age, gender, and stage of cancer at diagnosis were not associated with FT. UK participants who expected to be on-treatment for the remainder of life or self-employed had 2.84 times (95% CI 1.04–7.77) or 2.45 times (95% CI 1.04–5.73) greater odds of FT/more severe FT compared to participants who were off-treatment or employed full-time, respectively. In contrast, being UK retirees or the main earner led to 87% (adjusted OR (aOR) = 0.13, 95% CI 0.04–0.41) or 60% (aOR = 0.4, 95% CI 0.21–0.75) decrease in the odds of FT/more severe FT compared to those employed full-time or were not the main earner, respectively. Higher income was associated with a 72–95% decrease in the odds of facing FT/more severe FT (Table 3).

**Table 3:** Results of the ordered logistic model investigating FT and its associated factors in the UK

	Severity of financial toxicity	
	Adjusted odd ratios (aOR)^§^	95% CI
Age group		
18–64 years old^ref^	1.00	.
65 + years old	1.19	[0.44, 3.23]
Gender		
Male^ref^	1.00	.
Female	0.83	[0.44, 1.57]
Occupation		
Employed full-time^ref^	1.00	.
Employed part-time	0.91	[0.40, 2.10]
Unemployed	0.81	[0.19, 3.42]
Self-employed	2.45*	[1.04, 5.73]
Full-time homemaker	1.70	[0.38, 7.56]
Retired	0.13**	[0.04, 0.41]
Still studying	0.43	[0.02, 7.88]
Disabled/too ill to work	6.40**	[2.28, 17.95]
Main earner		
No^ref^	1.00	.
Yes	0.40^*^	[0.21, 0.75]
Household weekly income (before tax)		
Up to £200 (up to $250)^ref^	1.00	.
£200–399 ($250–499)	0.56	[0.18, 1.69]
£400–599 ($500–749)	0.36	[0.12, 1.12]
£600–799 ($750–999)	0.28^*^	[0.08, 0.95]
£800–999 ($1000–1249)	0.05^**^	[0.01, 0.24]
£1000–1199 ($1250–1499)	0.15^*^	[0.04, 0.63]
£1200–1399 ($1500–1749)	0.13^*^	[0.03, 0.59]
£1400 or above ($1750 or above)	0.07^**^	[0.02, 0.31]
Stage of cancer at diagnosis		
Stage 0 (Carcinoma in situ)^ref^	1.00	.
Stage I	0.75	[0.24, 2.30]
Stage II	0.86	[0.29, 2.58]
Stage III	1.40	[0.46, 4.29]
Stage IV (metastatic)	2.55	[0.72, 9.06]
Treatment status		
Off-treatment (in remission/N.E.D)^ref^	1.00	.
On-treatment	1.76	[0.83, 3.76]
Expected to be on-treatment for the remainder of life	2.84^*^	[1.04, 7.77]

*CI* confidence interval, *N.E.D* no evidence of disease state

^ref^Reference group

^§^Exponentiated coefficients

^*^p < 0.05 vs reference group; **p < 0.001 vs reference group

In the USA/US, main earner status, stage of cancer at diagnosis, and treatment status (on-treatment/off-treatment/expected to be on-treatment for the remainder of life) were not associated with FT. USA/US female or part-time employed participants had 1.83 times (95% CI 1.01–3.32) or 2.32 times (95% CI 1.02–5.26) greater odds of FT/more severe FT compared to male and full-time employed participants, respectively. In contrast, being USA/US retirees or aged 65 + years led to 74% (aOR = 0.26, 95% CI 0.09–0.75) or 81% (aOR = 0.19, 95% CI 0.07–0.48) decrease in the odds of experiencing FT/more severe FT compared to those employed full-time or were 18–64 years old, respectively. Higher income led to 84–95% decrease in the odds of facing FT/more severe FT (Table 4).

**Table 4:** Results of the ordered logistic model investigating FT and its associated factors in the USA/US

	Severity of financial toxicity	
	Adjusted odd ratios (aOR)^§^	95% CI
Age group		
18–64 years old^ref^	1.00	.
65 + years old	0.19^**^	[0.07, 0.48]
Gender		
Male^ref^	1.00	.
Female	1.83^*^	[1.01, 3.32]
Occupation		
Employed full-time^ref^	1.00	.
Employed part-time	2.32^*^	[1.02, 5.26]
Unemployed	1.13	[0.32, 3.95]
Self-employed	0.31^*^	[0.12, 0.78]
Full-time homemaker	1.18	[0.31, 4.45]
Retired	0.26^*^	[0.09, 0.75]
Still studying	0.86	[0.17, 4.42]
Disabled/too ill to work	3.07	[0.99, 9.47]
Main earner		
No^ref^	1.00	.
Yes	0.84	[0.44, 1.57]
Household weekly income (before tax)		
Up to £200 (up to $250)^ref^	1.00	.
£200–399 ($250–499)	0.16^*^	[0.04, 0.55]
£400–599 ($500–749)	0.34	[0.10, 1.09]
£600–799 ($750–999)	0.19^*^	[0.05, 0.68]
£800–999 ($1000–1249)	0.13^*^	[0.03, 0.53]
£1000–1199 ($1250–1499)	0.19^*^	[0.05, 0.76]
£1200–1399 ($1500–1749)	0.03^**^	[0.01, 0.14]
£1400 or above ($1750 or above)	0.05^**^	[0.02, 0.18]
Stage of cancer at diagnosis		
Stage 0 (carcinoma in situ)^ref^	1.00	.
Stage I	0.84	[0.35, 1.99]
Stage II	1.52	[0.62, 3.70]
Stage III	1.62	[0.60, 4.40]
Stage IV (metastatic)	2.35	[0.60, 9.12]
Treatment status		
Off-treatment (in remission/N.E.D)^ref^	1.00	.
On-treatment	1.66	[0.78, 3.53]
Expected to be on-treatment for the remainder of life	2.29	[0.83, 6.28]

*CI* confidence interval, *N.E.D* no evidence of disease state

^ref^Reference group

^§^Exponentiated coefficients

^*^p < 0.05 vs reference group; **p < 0.001 vs reference group

## Discussion

### Prevalence of FT in the USA/US and UK

The prevalence of FT in the USA/US was 1.5–2 times higher than that of the UK. The result is, perhaps, unsurprising as the two countries have different health financing systems; the latter has a publicly funded health system that seems to provide a greater degree of financial protection than that offered in the US. However, it is not a full protection as 33% of UK cancer patients and survivors still faced FT during their cancer journey.

Our findings regarding FT are similar to studies from other countries with publicly funded health system and universal health coverage. In Germany, a study of 100 patients who received radiotherapy reported that 31% suffered subjective financial distress which was significantly associated with active employment, lower household income, higher direct costs, and higher loss of income [11]. In Italy, a study with pooled data from 16 prospective multicentre trials reported 22.5% patients experienced FT [5]. In Canada, a review found that 38–71% of cancer patients faced financial distress which was associated with the household’s financial status, competency in managing finances, and lost wages [12]. Studies from countries with publicly funded health system but using co-payment also reported the existence of FT. In Japan, where co-payment ranges from 10 to 30%, a study of 190 patients who were receiving chemotherapy found that 56% experienced moderate or severe FT which was significantly associated with income-related factors [10]. In Vietnam, where co-payment is 20%, 41% of breast cancer patients experienced FT [4]. Although these figures from different countries cannot be compared directly to each other as they all used different tools to measure FT, the role of the health system financing is consistent.

Findings from this study are also consistent with previous studies in the USA/US [23, 24] and UK [25, 26] about financial distress and the use of savings or life’s assets to cover costs related to treatment. Direct comparison can be made possible with a USA/US study which also used COST to measure FT amongst 308 patients with gynaecologic cancer and reported that 47% faced FT [27]. This is somewhat lower than the prevalence of 55% observed in our study though it is noted that this study covered more types of cancer including those that are more expensive to treat such as lung, breast, colorectal, leukaemia, and blood cancer.

### Distribution of FT amongst sociodemographic groups in the USA/US and UK

The USA/US had significantly higher FT prevalence than the UK in almost every sociodemographic groups. The exceptions, therefore, provide some insight into how the risk of FT might be reduced.

Firstly, prevalence of FT amongst those who were on-treatment or off-treatment (i.e. in remission/N.E.D) in the UK was significantly lower than that of the USA/US but amongst the group who expected to receive treatment for the remainder of life (or life-time patients), the difference in prevalence was not observed. Perhaps, this relates to changes in the nature of expenses and the supports offered in systems. For example, whilst in the UK healthcare may provide comprehensive cover of treatment, it does not offer longer term protection for personal services, home adaptations, changed living costs, changes to earning capacity, or the full costs of palliative care. Further research on how to reduce FT for life-time patients, therefore, is needed.

Whilst FT prevalence amongst USA/US participants aged 18–64 years were nearly doubled that of the UK, there was no significant difference in FT prevalence between the US and UK amongst group 65 + years old. Furthermore, US participants aged 65 + years had 81% decrease in the odds of facing more severe FT compared to those 18–64 years old, whilst age did not play a significant role in the UK. This result is similar with previous study in the US wherein proportion of cancer survivors aged 65 + faced financial hardship was significantly higher than that of those aged 18–64 years old (53.6% vs 42%, respectively) [28]. This might be explained by the availability of Medicare for those 65 + years old in the USA/US. Previous studies showed that Medicare and age 65 + years old were associated with decreased cancer-related financial problems [29, 30]. Likewise, the protection afforded by Medicare for USA/US citizens who experience cancer which is more akin to the universal coverage afforded all UK citizens could also explain why there was no significant difference in FT prevalence between the USA/US and UK amongst group of retirees. Nevertheless, further research with data on the health insurance possessed benefits acquired during cancer journey of patients and survivors facing FT may shed more light on this.

### Associated factors with FT in the USA/US and UK

In the UK, age, sex, and stage of cancer at diagnosis were not associated with FT. These findings may be explained by the free at point of use cancer treatment available for all UK residents. Previous studies reported that households, on average, were £570/month worse off following a diagnosis of cancer [25]; the parents of a young child with cancer or the families of young adult cancer patients spent an extra £600 or £360/month, respectively [31]. This may explain why those with higher income had 72–95% decrease in the odds of facing more severe FT. Self-employed individuals, in contrast, were more vulnerable as they may have been at greater risk of losing income by not being able to work compared to full-time employees (i.e. supported by sick leave) or retirees (i.e. entitled to state and/or private pension). Loss of income plus additional costs may increase the likelihood (2.45 times) of experiencing more severe FT amongst self-employed participants.

In the USA/US, by contrast, part-time employees were the most vulnerable to FT and were 2.32 times more likely to experience severe FT that those in other employment categories. Being retired or having a higher income was found to lower the risk of FT. Interestingly, women were more likely to experience severe FT than men. These findings are consistent with previous studies in USA/US [8, 32] highlighting a greater vulnerability for women that may relate in part to the labour market, in part to the division of household responsibilities, and in part to the cancers experienced. If women disproportionately occupy jobs with poor insurance cover/sickness benefits, are more likely to work part-time, and/or are more likely to experience longer cancer journeys, collectively, it may make them more vulnerable in a USA/US type system to FT. Further research in this area could also usefully be undertaken.

### Strength and limitations

To the best of our knowledge, this is the first comparative study that investigated the prevalence of FT in the USA/US and UK. The use of a widely recognised and validated instrument is a strength of the study and its use facilitated analytical comparisons. The paper highlights the high prevalence of FT in both countries despite the existence of greater public funding of treatment in the UK. The paper draws attention to differences in the experience of FT that may be particularly helpful for interventions. Despite its strengths, the study has some limitations.

Firstly, this is a cross-sectional study; therefore, whether causal relationships between sociodemographic variables, clinical characteristics, and the occurrence or severity of FT exist cannot be inferred. A prospective cohort study is required to investigate causal relationships. Second, due to the use of convenience sampling, the sample may not be representative of the USA/US and the UK and the extent to which study results may be generalised is unclear. Further research with representative samples is required. Third, we could not explore the potential role or influence of race/ethnicity and cancer type regarding the prevalence of FT because the sample comprised mainly White participants, and whilst several types of cancer were captured, the number of participants by cancer site was small such that subgroup analysis by cancer type was not possible. Fourth, significant geographic heterogeneity may exist within countries as well as between them related, inter alia, to labour market conditions and the availability of government supports. Further research could usefully examine these issues.

## Conclusions

This cross-sectional survey of cancer patients and survivors in the USA/US and UK demonstrates that FT is very common, although FT prevalence and severity were worse in the US, compared to the UK. Distinct patterns in the risk of FT also exist between the two countries. The differences in FT prevalence and its risk factors are likely grounded in the differential protection afforded by healthcare funding models in the USA/US and UK. Nevertheless, the fact that both the USA/US and UK cancer patients and survivors are facing severe FT underscores the need for additional protections than are currently offered in either country to the most vulnerable groups such as those were younger than 65 years old, life-time patients, women, self-employed/employed part-time, or having low income.

## Supplementary Information

Below is the link to the electronic supplementary material.

Supplementary file1 (DOCX 35 KB)

## Data Availability

The dataset underlying this article cannot be shared due to the restrictions in participant’s informed consent “All information that you provide will remain confidential and will be accessible only to the researchers conducting this study. Data collected during the study may be published in academic journals and presented at conferences. Such data will be anonymised and presented in aggregated and/or summarised form”. All aggregated and/or summarised data were presented in this paper and its supplementary information.
