# Supplementary material for: Financial toxicity amongst cancer patients and survivors: a comparative study of the United Kingdom and United States
Source: Support Care Cancer. 2025 May 31;33(6):521. doi: 10.1007/s00520-025-09568-6 (PMC12126310; doi:10.1007/s00520-025-09568-6)
Supplement: Supplementary file 1 — Supplementary file1 (DOCX 35 KB) [file 520_2025_9568_MOESM1_ESM.docx]

**Supplementary Table 1. COmprehensive Score for financial Toxicity (COST) items and scoring guidelines**

**COmprehensive Score for financial Toxicity (COST) items**

Below is a list of statements that other people with your illness have said are important. Please circle or mark one number per line to indicate your response as it applies to the past 7 days.

|  |  | **Not at all** | **A little bit** | **Some-what** | **Quite**  **a bit** | **Very much** |
| --- | --- | --- | --- | --- | --- | --- |
| FT1 | I know that I have enough money in savings, retirement, or assets to cover the costs of my treatment | 0 | 1 | 2 | 3 | 4 |
| FT2 | My out-of-pocket medical expenses are more than I thought they would be | 0 | 1 | 2 | 3 | 4 |
| FT3 | I worry about the financial problems I will have in the future as a result of my illness or treatment | 0 | 1 | 2 | 3 | 4 |
| FT4 | I feel I have no choice about the amount of money I spend on care | 0 | 1 | 2 | 3 | 4 |
| FT5 | I am frustrated that I cannot work or contribute as much as I usually do | 0 | 1 | 2 | 3 | 4 |
| FT6 | I am satisfied with my current financial situation | 0 | 1 | 2 | 3 | 4 |
| FT7 | I am able to meet my monthly expenses | 0 | 1 | 2 | 3 | 4 |
| FT8 | I feel financially stressed | 0 | 1 | 2 | 3 | 4 |
| FT9 | I am concerned about keeping my job and income, including work at home | 0 | 1 | 2 | 3 | 4 |
| FT10 | My cancer or treatment has reduced my satisfaction with my present financial situation | 0 | 1 | 2 | 3 | 4 |
| FT11 | I feel in control of my financial situation | 0 | 1 | 2 | 3 | 4 |
| FT12 | My illness has been a financial hardship to my family and me | 0 | 1 | 2 | 3 | 4 |

**COmprehensive Score for financial Toxicity (COST) Scoring Guidelines** (Version 2)

Instructions:* 1. Record answers in "item response" column. If missing, mark with an X

2. Perform reversals as indicated, and sum individual items to obtain a score.

3. Multiply the sum of the item scores by the number of items in the scale, then divide by the

number of items answered. This produces the scale score.

4. **The higher the score, the better the Financial Well-Being.**

**Subscale Item Code Reverse item? Item response Item Score**

**FINANCIAL** FT1 0 + ________ =________

**TOXICITY** FT2 4 - ________ =________

**SCALE** FT3 4 - ________ =________

FT4 4 - ________ =________

FT5 4 - ________ =________

*Score range:* 0-44

FT6 0 + ________ =________

FT7 0 + ________ =________

FT8 4 - ________ =________

FT9 4 - ________ =________

FT10 4 - ________ =________

FT11 0 + ________ =________

FT12 **Not scored (summary item)**

***Sum individual item scores:*** ________

***Multiply by 11:*** ________

**Financial Toxicity Score**

***Divide by number of items answered:*** **________*=***

**Supplementary Table 2. Participants’ living area in the United Kingdom and United States**

| **12 regions of United Kingdom (n=319)** | **n** | **%** |
| --- | --- | --- |
| Scotland | 26 | 8.2 |
| North East | 8 | 2.5 |
| North West | 46 | 14.4 |
| Yorkshire and Humberside | 37 | 11.6 |
| East Midlands | 30 | 9.4 |
| Wales | 14 | 4.4 |
| South East | 49 | 15.4 |
| Northern Ireland | 6 | 1.9 |
| East Anglia | 26 | 8.2 |
| South West | 27 | 8.5 |
| West Midlands | 32 | 10.0 |
| London | 18 | 5.6 |
| **9 divisions of the United States* (n=281)** |  |  |
| New England | 15 | 5.3 |
| Middle Atlantic | 36 | 12.8 |
| East North Central | 40 | 14.2 |
| West North Central | 11 | 3.9 |
| South Atlantic | 61 | 21.7 |
| East South Central | 25 | 8.9 |
| West South Central | 33 | 11.7 |
| Mountain | 23 | 8.2 |
| Pacific | 37 | 13.2 |
| ** States in each divisions are as follows*  *New England: Connecticut, Maine, Massachusetts, New Hampshire, Rhode Island, Vermont*  *Middle Atlantic: New Jersey, New York, Pennsylvania*  *East North Central: Indiana, Illinois, Michigan, Ohio, Wisconsin*  *West North Central: Iowa, Kansas, Minnesota, Missouri, Nebraska, North Dakota, South Dakota*  *South Atlantic: Delaware, District of Columbia, Florida, Georgia, Maryland, North Carolina, South Carolina, Virginia, West Virginia*  *East South Central: Alabama, Kentucky, Mississippi, Tennessee*  *West South Central: Arkansas, Louisiana, Oklahoma, Texas*  *Mountain: Arizona, Colorado, Idaho, New Mexico, Montana, Utah, Nevada, Wyoming*  *Pacific: Alaska, California, Hawaii, Oregon, Washington* | | |

**Supplementary Table 3. Participant’ type of cancer**

| **Type of cancer, n (%)** | **United Kingdom**  **n (%)** | **United States**  **n (%)** | **Total**  **n (%)** |
| --- | --- | --- | --- |
| Breast cancer | 99 (31.0) | 79 (28.1) | 178 (29.7) |
| Lung cancer | 5 (1.6) | 3 (1.1) | 8 (1.3) |
| Prostate cancer | 17 (5.3) | 10 (3.6) | 27 (4.5) |
| Bowel/Colorectal cancer | 23 (7.2) | 18 (6.4) | 41 (6.8) |
| Skin cancer | 34 (10.7) | 26 (9.3) | 60 (10.0) |
| Hodgkin/Non-Hodgkin lymphoma | 24 (7.5) | 20 (7.1) | 44 (7.3) |
| Kidney | 8 (2.5) | 6 (2.1) | 14 (2.3) |
| Head and neck | 24 (7.5) | 24 (8.5) | 48 (8.0) |
| Leukemia, blood cancer | 17 (5.3) | 14 (5.0) | 31 (5.2) |
| Bone cancer | 8 (2.5) | 3 (1.1) | 11 (1.8) |
| Cervical cancer | 12 (3.8) | 15 (5.3) | 27 (4.5) |
| Uterine and endometrial cancer | 9 (2.8) | 17 (6.0) | 26 (4.3) |
| Ovarian cancer | 14 (4.4) | 10 (3.6) | 24 (4.0) |
| Penile/testicular cancer | 16 (5.0) | 10 (3.6) | 26 (4.3) |
| Other | 9 (2.8) | 26 (9.3) | 35 (5.8) |
| **Total** | **319 (53.2)** | **281 (46.8)** | **600 (100.0)** |
